# Transcriptional and open chromatin analysis of bovine skeletal muscle development by single‐cell sequencing

**DOI:** 10.1111/cpr.13430

**Published:** 2023-03-01

**Authors:** Cuicui Cai, Peng Wan, Hui Wang, Xin Cai, Jiabo Wang, Zhixin Chai, Jikun Wang, Haibo Wang, Ming Zhang, Nan Yang, Zhijuan Wu, Jiangjiang Zhu, Xueyao Yang, Yulian Li, Binglin Yue, Ruihua Dang, Jincheng Zhong

**Affiliations:** ^1^ Key Laboratory of Animal Genetics, Breeding and Reproduction of Shaanxi Province, College of Animal Science and Technology Northwest A&F University Yangling China; ^2^ Guyuan Branch Ningxia Academy of Agriculture and Forestry Sciences Guyuan China; ^3^ Key Laboratory of Qinghai‐Tibetan Plateau Animal Genetic Resource Reservation and Utilization, Sichuan Province and Ministry of Education Southwest Minzu University Chengdu China

## Abstract

Skeletal muscle is a complex heterogeneous tissue and characterizing its cellular heterogeneity and transcriptional and epigenetic signatures are important for understanding the details of its ontogeny. In our study, we applied scRNA‐seq and scATAC‐seq to investigate the cell types, molecular features, transcriptional and epigenetic regulation, and patterns of developing bovine skeletal muscle from gestational, lactational and adult stages. Detailed molecular analyses were used to dissect cellular heterogeneity, and we deduced the differentiation trajectory of myogenic cells and uncovered their dynamic gene expression profiles. SCENIC analysis was performed to demonstrate key regulons during cell fate decisions. We explored the future expression states of these heterogeneous cells by RNA velocity analysis and found extensive networks of intercellular communication using the toolkit CellChat. Moreover, the transcriptomic and chromatin accessibility modalities were confirmed to be highly concordant, and integrative analysis of chromatin accessibility and gene expression revealed key transcriptional regulators acting during myogenesis. In bovine skeletal muscle, by scRNA‐seq and scATAC‐seq analysis, different cell types such as adipocytes, endothelial cells, fibroblasts, lymphocytes, monocytes, pericyte cells and eight skeletal myogenic subpopulations were identified at the three developmental stages. The pseudotime trajectory exhibited a distinct sequential ordering for these myogenic subpopulations and eight distinct gene clusters were observed according to their expression pattern. Moreover, specifically expressed TFs (such as MSC, MYF5, MYOD1, FOXP3, ESRRA, BACH1, SIX2 and ATF4) associated with muscle development were predicted, and likely future transcriptional states of individual cells and the developmental dynamics of differentiation among neighbouring cells were predicted. CellChat analysis on the scRNA‐seq data set then classified many ligand–receptor pairs among these cell clusters, which were further categorized into significant signalling pathways, including BMP, IGF, WNT, MSTN, ANGPTL, TGFB, TNF, VEGF and FGF. Finally, scRNA‐seq and scATAC‐seq results were successfully integrated to reveal a series of specifically expressed TFs that are likely to be candidates for the promotion of cell fate transition during bovine skeletal muscle development. Overall, our results outline a single‐cell dynamic chromatin/transcriptional landscape for normal bovine skeletal muscle development; these provide an important resource for understanding the structure and function of mammalian skeletal muscle, which will promote research into its biology.

## Graphical abstract

Cai et al. applied single‐cell RNA sequencing (scRNA‐seq) and single‐cell assay for transposase‐accessible chromatin sequencing (scATAC‐seq) to investigate the cell types, molecular features, transcriptional and epigenetic regulation, and patterns of developing bovine skeletal muscle from gestational, lactational and adult stages. Detailed molecular analysis was used to dissect cellular heterogeneity, and the differentiation trajectory of myogenic cells was deduced to uncover their dynamic gene expression profiles. SCENIC analysis was performed to demonstrate key regulons during cell fate decisions. The future expression state of these heterogeneous cells was explored by RNA velocity analysis and extensive networks of intercellular communication were analysed using the toolkit CellChat. Moreover, the transcriptomic and chromatin accessibility modalities were confirmed to be highly concordant and integrative analysis of chromatin accessibility and gene expression revealed key transcriptional regulators acting during myogenesis.

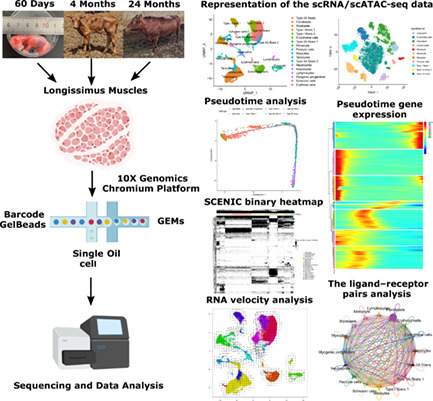

## 1 INTRODUCTION

Skeletal muscle maintains basic mammalian body functions such as metabolism, respiration and locomotion. It is composed of a mixture of multinucleated myofibers, muscle stem cells, endothelial cells, immune cells, adipocytes, neurocytes and other mononuclear cells.^1^ The formation, growth, and maintenance of skeletal muscle in vertebrates require the successive phases of foetal, postnatal and adult myogenesis, which are controlled by autonomous cell pathways and cell–cell communication within the developing skeletal muscle. In general, the main waves of myogenesis occur during development in utero, when myogenic progenitor cells undergo specification, determination and differentiation to form mature myofibers. After birth, although the number of myofibers remains constant, satellite cells can be activated to re‐enter proliferation and differentiation programmes to replace damaged muscle.^2^ These processes are regulated by some known signal molecules from surrounding cells and are coordinated precisely by transcription factors such as Pax3, Pax7, MRFs and MEF2 family.^3^, ^4^, ^5^ However, the relationship between myogenic cells and neighbouring cell types has received much less attention. Thus, given their varied molecular and functional states, our understanding of skeletal muscle development remains incompletely defined. It is believed that different cells within skeletal muscle establish efficient communication strategies to allow the exchange of biological information and reciprocal dialogues among these cells are crucial for muscle homeostasis and function. Accordingly, it is important to understand the functional capacities and responses of each cell type in skeletal muscle.

Currently, the application of scRNA‐seq permits characterization of transcriptomes at the single‐cell level at different development stages. The most comprehensive single‐cell sequencing data on skeletal muscle development comes from the study by Xi et al. based on specific marker genes, the authors identified and visualized various cell populations present during human limb skeletal muscle development.^6^ This makes it possible to assess distinct cell types and states, their dynamic trajectories and molecular programmes governing sequential cell fates in skeletal muscle development.^7^ Moreover, recent advances in scATAC‐seq offer a single‐cell method for measuring chromatin accessibility to generate additional information about gene regulatory processes. Combined with scRNA‐seq, these approaches have the potential to unravel how dynamic changes in cis‐regulatory elements driven by changes in TF binding regulate gene expression programmes during development.^8^, ^9^ A recent study established an atlas of the gene expression pattern and transcriptional regulation on mouse limb skeletal muscle by combining scRNA‐seq/scATAC‐seq and FISH experiments, in which, myonuclei of slow and fast myofibers, myotendinous junction (MTJ) and neuromuscular junction (NMJ) with specific transcriptional programmes were identified within a myofiber.^10^ Yet myogenic development in bovine tissues at the single‐cell level is poorly understood. Here, to resolve gene‐regulatory dynamics during bovine skeletal muscle ontogeny, we generated gene expression profiles and chromatin accessibility at single‐cell resolution from bovine *longissimus dorsi* muscle samples spanning gestational, lactational and adult stages. We revealed myogenic cells and neighbouring cell types to be present at distinct developmental stages and defined a developmental trajectory for myogenic cells, delineating their cellular state transitions and intercellular communication, as well as key regulators underlying these processes. In addition, a continuous progression of TF motif activities associated with myogenesis was revealed and an integrative analysis of chromatin accessibility and gene expression revealed key transcriptional regulators acting during myogenesis. Overall, our work provides a model of transcriptional and epigenetic regulations and delineates central transcriptional, metabolic and signalling pathways during bovine skeletal muscle ontogeny at single‐cell resolution.

## 2 MATERIALS AND METHODS

### 2.1 Animals

Longissimus muscle samples of Qinchuan cattle were collected from foetal (60 days), postnatal (4 months) and adult (24 months) stages at Ningxia Yikang Animal Husbandry. The cow was artificially inseminated on natural oestrus. All the cattles were not fed the night before they were euthanized. The care and use of experimental animals were in full compliance with local animal welfare laws, guidelines and policies.

### 2.2 scRNA‐seq data processing

The 10× Genomics scRNA‐seq raw data were processed and aligned to the reference genome downloaded from ENSEMBL (release version 104) and then quantified using the package Cell Ranger (release 6.0.2). Then, we utilized the R package Seurat (release 4.0.5) to perform downstream analysis.^9^ First, cells with low detected gene numbers, low total read numbers and high ratios of mitochondrial to somatic genes were detected automatically using the R function ‘isOutlier' and then filtered out. Then, the count matrix was normalized and scaled using the ‘NormalizeData’ and ‘ScaleData’ functions. We performed principal component analysis (PCA) based on the top 2000 highly variable genes using the ‘RunPCA’ function. To reduce background noise, the significant PCA results were selected according to the *p* values generated by ‘ScoreJackStraw’. We clustered cells using the ‘FindClusters’ function and then projected them onto a two‐dimensional space using the ‘RunTSNE’ or ‘RunUMAP’ functions.

### 2.3 Integrative analysis of scRNA‐seq data

Before integrating multiple scRNA‐seq data from the three different developmental stages, we first examined whether there were any batch effects. We found that the strongest remaining factor was sample‐to‐sample differences, because cells apparently tended to cluster by sample. Therefore, we applied the R package ‘Harmony’ (release 0.1.0) to correct batch effects.^11^ After that, we annotated cell clusters by first identifying the marker genes of each cluster with the ‘FindMarkers’ function from the R Seurat toolkit and then manually annotating the cell clusters according to known cell markers for each cell type. Clusters that expressed the same marker genes were merged.

### 2.4 Trajectory analysis of scRNA‐seq and scATAC‐seq data

To infer the developmental trajectory of particular cell lineages, we used the R package ‘Monocle2’ (release 2.20.0) to perform trajectory analysis based on scRNA‐seq and scATAC‐seq data.^12^ Specifically, the differentially expressed genes between different cell types were selected for following analyses. Next, the ‘DDRTree’ method was applied to perform dimensionality reduction and order the cells along pseudotime. Because Monocle2 cannot know a priori which of the trajectory of the tree to call the ‘beginning’, the ‘root_state’ argument was used to specify the zygote stage as the beginning and we then reapplied ‘orderCells’.

### 2.5 Single‐cell regulatory network inference

To infer the regulatory activity of TFs in subpopulations, we used the pySCENIC package (release 0.11.2) to perform gene regulatory network analysis.^13^ Briefly, regulatory modules were identified by inferring coexpression between TFs and genes containing TF‐binding motifs in their promoter regions. First, we generated two gene‐motif ranking files including 10 kb around the TSS and 500 bp upstream. Then the ‘GRNBoost’ function implemented in pySCENIC was employed to identify coexpression modules and quantify the weight between TFs and target genes. Target genes with a low positive correlation (<0.03) in each TF module were removed from further analysis. The regulatory activity of TFs was quantified by area under the recovery curve value from the enrichment of each regulon. Subpopulation‐specific regulons were identified according to the average regulon activity scores in the subpopulation.

### 2.6 RNA velocity analysis

To infer the developing trajectory of individual cells or cell populations, we employed the ‘velocyto’ (package release 0.17.17) to estimate RNA velocities of single cells by quantifying unspliced and spliced mRNAs based on output files from the package ‘Cell Ranger'.^14^ The spliced/unspliced expression matrices of genes were processed using the R package ‘velocyto.R’ (release 0.6) to estimate velocity. Then, we visualized RNA velocity by projecting gene unspliced/spliced reads abundance on the clustering embedding space generated by integrative analysis.

### 2.7 Cell communication analysis

To quantify intercellular communication networks, we used the ‘CellChat’ package (release 1.1.3) to investigate potential cell communications.^15^ Because there is incomplete annotation of receptor–ligand pair information for the bovine geneome, we only selected homologous human genes for further analyses. Briefly, the CellChat objects were first created using the ‘createCellChat’ function. Then, the communication probability was inferred using ‘computeCommunProb’ and ‘computeCommunProbPathway’ functions. The network centrality scores and the contribution of each ligand–receptor pair to the signalling pathway were calculated using ‘netAnalysis_computeCentrality’ and ‘netAnalysis_contribution’, respectively. All graphs of visualized cell communications were generated using the functions implemented in the CellChat package.

### 2.8 Single‐cell ATAC‐seq (scATAC‐seq) analysis pipeline

#### 2.8.1 Pre‐processing

The fastq files of scATAC‐seq data were first processed by the function ‘cellranger‐atac count’ of 10× Genomics Cell Ranger ATAC (version 2.0.0) pipeline, which generated fragment files and per‐barcode fragment metrics after filtering the reads, alignment, barcode counting, identification of transposase cut sites and detection of accessible chromatin peaks. In the alignment step, we used the reference genome bosTau9.

#### 2.8.2 Creating cell‐by‐bin matrix

Downstream analyses were performed using the ‘SnapATAC’ (release 1.0.0) pipeline.^16^ Taking fragment files as input, we generated snap files by ‘snaptools’ (release 1.4.8), only considering uniquely aligned and properly paired fragments with a mapping quality ≥30. To create a cell‐by‐bin matrix, we segmented the genome into 5‐kb bins and scored each cell with the number of aligned fragments in a given bin using the fragment files. Only cells with high‐quality libraries were retained according to the total number of detected high‐quality fragments (1000–5000) and the ratio of high‐quality fragments overlapping with annotated transcription start sites (0.125–0.8). After binarization of the matrix, we calculated and normalized the coverage of each bin and finally eliminated those overlapping with invariant features and mitochondrial DNA to avoid potential contamination. Any cells with <500 bin coverages were discarded in the following analyses.

#### 2.8.3 Dimension reduction and cell clustering

Based on the cell‐by‐bin matrix, the Nyström landmark diffusion maps algorithm was applied for nonlinear dimensionality reduction after combining the samples of the three groups, which included sampling, embedding and extension. In short, we sampled 10,000 cells from the total as ‘landmarks’ in a density‐based manner developed in SCTransform and computed a diffusion map embedding. The remaining query cells were then projected onto the resulting low‐dimension embedding, finally generating a joint embedding space. Batch effects of three groups were corrected by R package Harmony (release 0.1.0).^11^ After that, using the top 20 significant components determined by an ad hoc method, a *k*‐nearest neighbour graph was constructed for cells clustering and grouped cells that were of the same type through a community finding algorithm.

#### 2.8.4 Calculation of gene activity scores

In preparing the integrating scATAC‐seq data and corresponding scRNA‐seq data for cell type annotation, we created a cell‐by‐gene matrix by calculating the number of fragments intersecting with the regions of genes body and 2 kb upstream from the TSS, which was performed using the function ‘createGmatFromMat’ in SnapATAC. In this matrix, we only selected the top 2000 highly variable genes of scRNA‐seq data for downstream analyses.

#### 2.8.5 scRNA‐seq‐based annotation

For cell type annotation of chromatin accessibility data, we followed the scRNA‐seq‐based annotation pipeline provided by SnapATAC.^16^ First, we converted the snap object into a Seurat object (release 4.0.5) which was integrated with the paired scRNA‐seq data based on the linkage between chromatin accessibility and gene expression in single cells.^9^ Cell types were predicted by calculating ‘prediction scores’ with the function ‘TransferData’. After that, ‘pseudo multi‐omics’ cells containing information of gene expression as well as chromatin accessibility were created by imputation of gene expression profile. At last, we re‐clustered cells with max prediction score larger than 0.5 using the first 20 dimensions and constructed TSNE graphs for visualization of the high‐dimensionality dataset.

#### 2.8.6 Identification of peaks and differentially accessible regions

We clustered fragments from cells of the same cell type and identified peaks using MACS2 (release 2.2.7.1) with the parameters ‘‐‐nomodel ‐‐shift 100 ‐‐ext 200 ‐‐qval 5 e‐2 ‐B –SPMR’.^17^ Peaks from all clusters were merged to create and add a cell‐by‐peak matrix to the snap object. Differentially accessible regions (DARs) for each cluster were recognized using function ‘findDAR’. In brief, for a given cluster of cells, snapATAC took their neighbouring cells or the remaining cells as background to perform differential analysis in line with the size of that cluster.^16^ Peaks with log‐transformed fold change >0 and FDR <0.05 were considered as significant DARs.

#### 2.8.7 GREAT analysis

Taking DARs as input, we used GREAT to infer candidate and active biological pathways in each cell cluster.^18^

#### 2.8.8 Motif enrichment analysis

TF motif enrichment analysis of DARs was performed using the ‘findMotifsGenome’ function of package HOMER (release 4.11) for identification of known and de novo motifs.^19^

#### 2.8.9 Integration with scRNA‐seq

To integrate the scATAC‐seq and scRNA‐seq data, we followed the multiway alignment using all pairs method tutorial embedded in package scAlign (release 1.8.0), which was achieved by associating the gene expression and gene activity scores.^20^ Seurat objects of scATAC‐seq and scRNA‐seq data were transformed into ‘SingleCellExperiment’ objects separately to create a combined scAlign object based on the top 15 components from canonical component analysis (CCA) and normalized gene expression values as well as activity scores of highly variable genes. The two data sets from the same samples were then aligned by means of the ‘scAlignMulti’ function built in scAlign, during which 64 latent dimensions were used. The resulting aligned cells were finally projected onto a low‐dimension embedding space using the top 25 dimensions of ‘ALIGNED.CCA’ through the ‘RunTSNE’ function.

### 2.9 Immunohistochemistry for PAX7 and MYH7

Immunohistochemistry was performed as previously described^21^ using rabbit polyclonal antibodies directed against PAX7 (Cell Signaling Technology) and MYH7 (Cell Signaling Technology). We performed immunohistochemical staining on the same paraffin‐embedded tissue blocks that were used for single‐cell sequencing. Before immunostaining, the paraffinized 5 μm sections of skeletal muscle were deparaffinized and submitted to antigen retrieval in Tris‐citrate buffer (Servicebio) followed by incubated with 3% hydrogen peroxide in phosphate‐buffered saline (PBS; Servicebio) to inhibit endogenous peroxidases. After closed with 10% bovine serum albumin (BSA; Servicebio), sections were incubated at 4°C overnight with primary antibodies followed by incubated 1 h with an HRP‐labelled secondary antibody (Cell Signaling Technology) at room temperature. Peroxidase activity was revealed with 3,3′‐diaminobenzidine (DAB; Servicebio), which produces a brown staining, and sections were then stained for 30 s with haematoxylin (Servicebio), dehydrated, and mounted. Finally, the slides were scanned under the E100 microscope (Nikon), and data were analysed using the Aipathwell software (Servicebio).

## 3 RESULTS

### 3.1 Identification of cellular heterogeneity during bovine skeletal muscle development using scRNA‐seq

**FIGURE 1:**
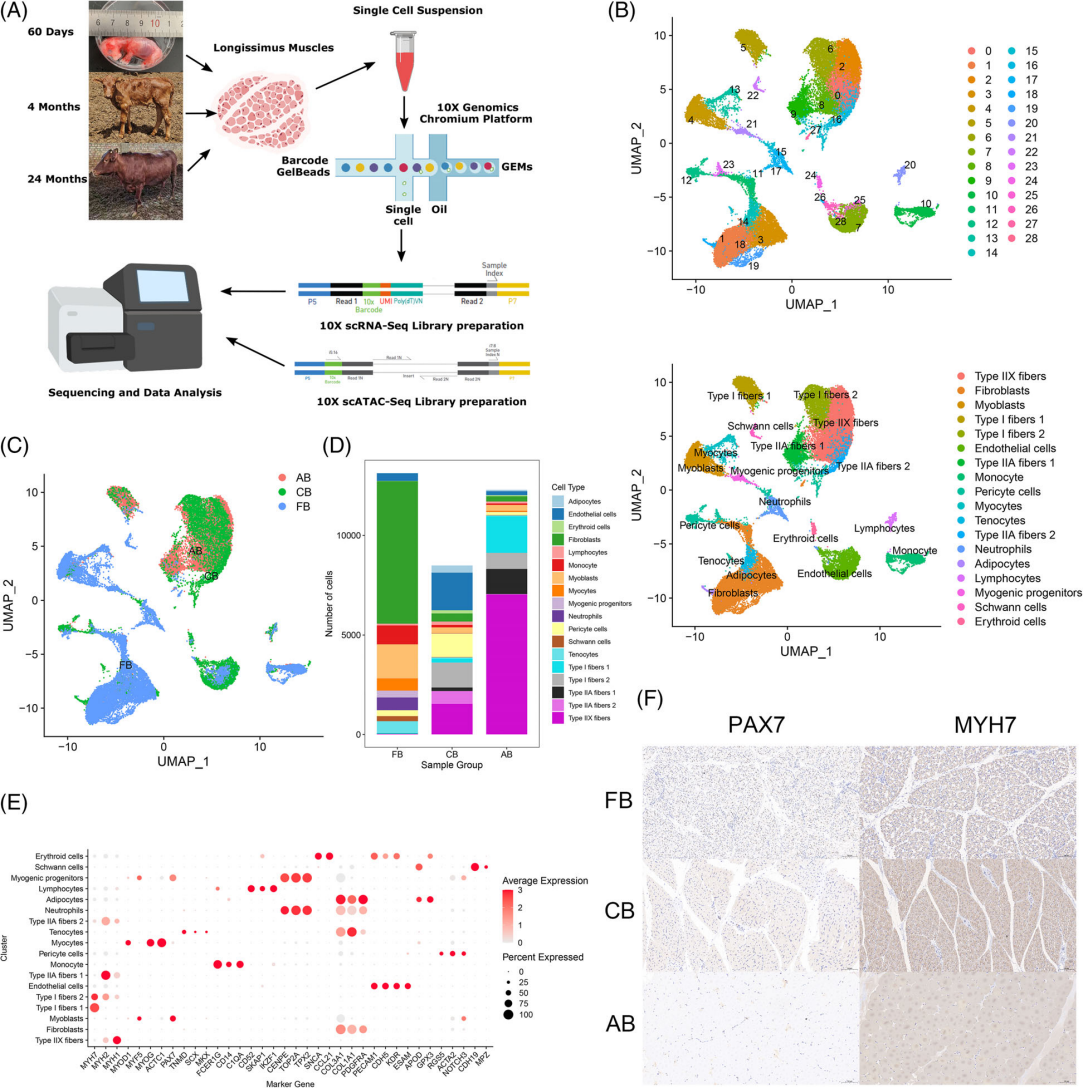
Single‐cell RNA sequencing reveals cellular heterogeneity during bovine skeletal muscle development. (A) Schematic diagram of the experimental procedure based on the 10× Chromium platform. Bovine skeletal muscle samples were analysed with scRNA‐seq and snATAC‐seq. (B) Twenty‐nine transcriptional cell clusters were revealed by unsupervised clustering and were projected on a UMAP plot (up), and UMAP plot of cells was coloured by cell type labelled with identity (down). Each point represents a single cell and is colour‐coded with cluster information. (C) UMAP plot of the integrated data set from three different time points. Cells are coloured according to developmental stage. AB, adult bovine; CB, calf bovine; FB, foetal bovine. (D) Histograms showing the percentage of transcriptionally defined cell types from the three stages. Colours represent the identified cell types. (E) This dot plot of the snRNA‐seq data set depicts the expression of cell‐type markers. The diameter of the dot represents the percentage of cells expressed and the colour intensity corresponds to the average expression level. APOD and GPX3 highly expressing adipocyte clusters (cluster 18); PECAM1, CDH5, KDR and ESAM highly expressing endothelial cell clusters (clusters 7, 25, 26 and 28); SNCA and CCL21 highly expressing erythroid cell clusters (cluster 24); COL3A1, COL1A1 and PDGFRA highly expressing fibroblast clusters (clusters 1, 3, 11, 19 and 27); CD52, SKAP1 and KZF1 highly expressing lymphocyte clusters (cluster 20); FCER1G, CD14 and C1QA highly expressing monocyte clusters (cluster 10); MYOD1 and MYF5 highly expressing myoblast clusters (cluster 4); MYOG and ACTC1 highly expressing myocyte clusters (cluster 13); PAX7 and MYF5 highly expressing myogenic progenitor cell clusters (cluster 21); CENPE, TOP2A and TPX2 highly expressing neutrophils clusters (cluster 17); RGS5, ACTA2 and NOTCH3 highly expressing pericyte cells (clusters 12, 15 and 23); CDH19 and MPZ highly expressing Schwann cell clusters (cluster 22); TNMD, SCX and MKX highly expressing tenocytes clusters (cluster 14); MYH7 highly expressing Type I fibres clusters (cluster 5 and 6); MYH2 highly expressing Type IIA fibre clusters (clusters 9 and 16); MYH1 highly expressing Type IIX fibre clusters (clusters 0, 2 and 8). (F) Immunohistochemical analysis of PAX7 and MYH7 expression in the bovine longissimus dorsi of FB, CB and AB. Scale bars, 100 μm.

To obtain a map of cell populations for the developing bovine skeletal muscle, *longissimus dorsi* tissues from foetal, postnatal and adult age were dissociated, and 43,398 single cells from these successive developmental stages (16,716, 10,100 and 16,582, respectively) were captured for performing droplet‐based scRNA‐seq (Figure 1A). After filtering out low‐quality metrics, we obtained 21,204 genes for foetal muscles, 19,644 genes for postnatal muscles and 20,863 genes for adult muscles, respectively. According to the UMAP protocol (https://umap-learn.readthedocs.io/en/latest/), 29 cell clusters were identified representing distinct cell populations at the three developmental stages (Figure 1B). To characterize the identity of each cell cluster, we evaluated the expression of lineage‐specific marker genes and identified 18 major cell types (Figure 1B,E). Thus, major cell populations in bovine *longissimus dorsi* during muscle development were identified successfully using these cell identity‐specific signature genes. Immunohistochemical analysis also indicated that PAX7 expressed in skeletal muscle of FB (foetal bovine) and CB (calf bovine), but not in AB (adult bovine), and MYH7 expressed in all three periods, further verifying the authenticity of our results (Figure 1F).

We also compared the proportions of cells from different developmental stages in each cluster, and observed remarkable changes in cell fractions between stages (Figure 1C,D). Notably, eight skeletal myogenic subpopulations were identified through UMAP analysis, in which, myoblasts and myogenic progenitors were mostly present at the foetal stage; and myofibers were largely present at postnatal and adult stages (Figure 1D). At age 60 days, the myogenic population in the developing muscle consisted mainly of *MYF5* ^+^ myoblasts and *MYOG* ^+^ myocytes, and a small subset of myogenic progenitors expressing *PAX7* was also observed (Figure 1D,E). Later, at the postnatal stage, several types of myofibers formed the majority of the myogenic population, with no differentiated myogenic cells detected, possibly because of the incorporation of most myogenic cells into multinucleated myofibers (Figure 1D,E). Adult skeletal muscles are composed mainly of slow and fast myofibers, which are classified as Type I fibres, Type IIA fibres and Type IIX fibres. In addition, Type IIB fibres were undetectable in our scRNA‐seq data, in line with the consensus that these genes are not expressed in skeletal muscles of large mammals such as humans and bovines.^22^, ^23^ Interestingly, hybrid myofibers expressing more than one *MYH* isoform were identified, in which Type I fibres 2 can express *MYH7* and *MYH2* while Type I fibres 1 only express *MYH7* (Figure 1E). For Type IIA fibres, one more *MYH7* copy is expressed in Type IIA fibres 2 than in Type IIA fibres 1. Analogously, using snRNA‐seq, mixed nuclei coexpressing *MYH7* with *MYH2* or *MYH1* with *MYH2* were detected in myofibers from adult mice, verified by RNAscope experiments.^10^ Furthermore, the percentages of Type I fibres 2 and Type IIA fibres 2 are higher than that of Type I fibres 1 and Type IIA fibres 1 at the postnatal stage; however, this trend is reversed at the adult stage. Myofibers are plastic and can adapt their contractile and metabolic properties based on the starting phenotype and the type of intervention by regulating *MYH* genes, in which fast *MYH* genes are located on a single locus while the slow *MYH7* gene lies on another locus. Recent studies have shown that two neighbouring genes can be expressed in the same nucleus at a given time through shared enhancers.^24^ Accordingly, exclusive expression could be controlled by a locus control region (such as the super enhancer), regulating the expression of associated foetal, postnatal and adult *MYH* genes in a temporal order. Nevertheless, myonuclear heterogeneity in syncytial myofibers remains unclear, but understanding this phenomenon will help illuminate the mechanisms controlling skeletal myogenesis.

### 3.2 Pseudotime patterns reveal the relationships among different myogenic subpopulations

**FIGURE 2:**
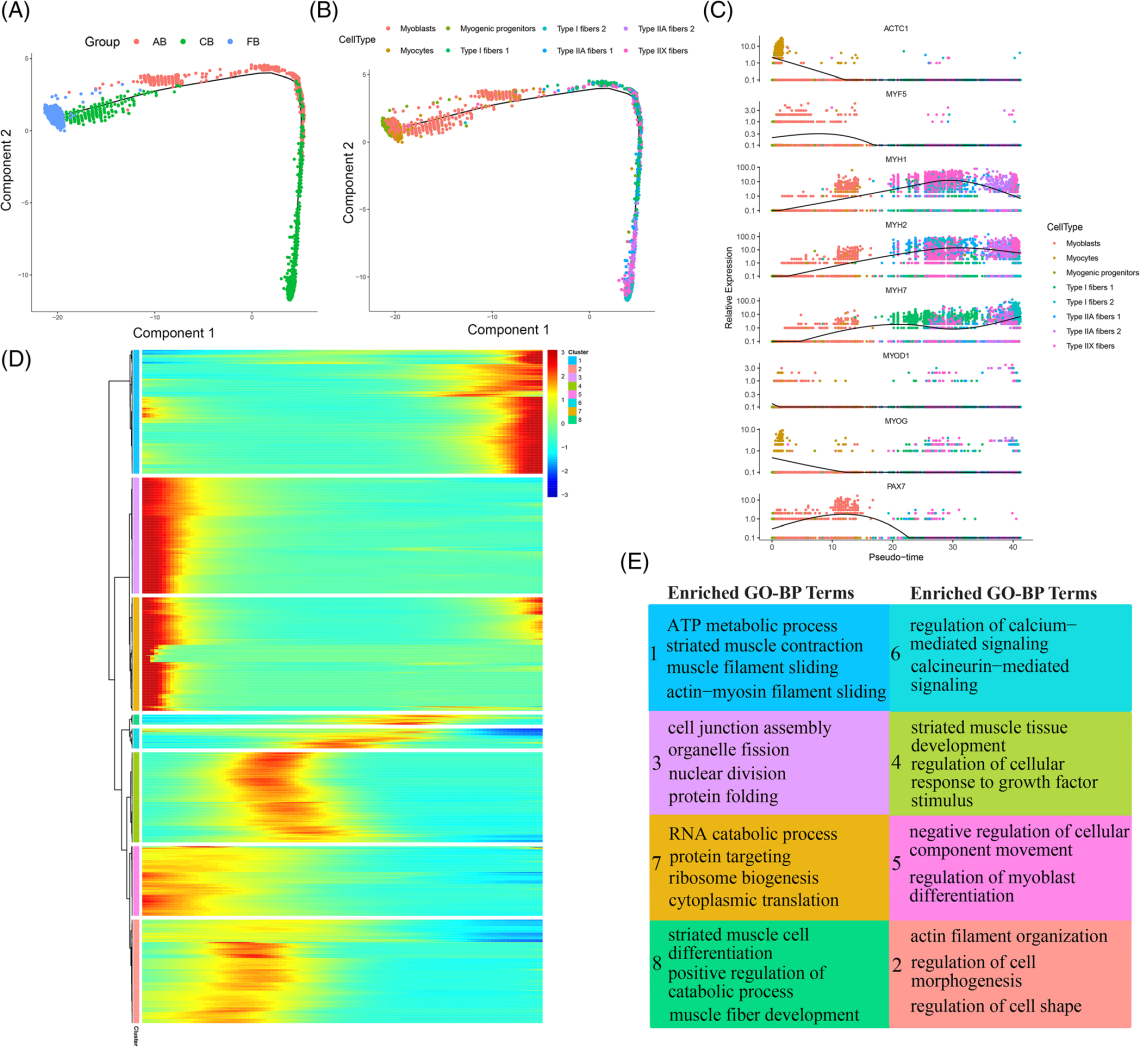
Differential gene expression profiles during bovine myogenesis. (A, B) Pseudotime analysis result of bovine skeletal myogenic subpopulations with Monocle2. Developmental time points and cell types are coloured based on pseudo‐time in (A) and (B) trajectory plots. (C) Expression levels (Scatter plot) of eight marker genes across pseudotime. The *x*‐axis represents pseudotime; the *y*‐axis indicates the normalized gene expression levels; while the colour refers to the eight myogenic subpopulations. (D) Eight clusters of pseudotime gene expressions are clustered hierarchically during bovine myogenesis. (E) Enriched GO–BP terms of the gene clusters.

To estimate the lineage relationships between the skeletal myogenic subpopulations, we performed pseudotime analysis of our single‐cell data on all skeletal myogenic clusters based on the Monocle2 algorithm. This algorithm permits one to order single cells along trajectories exhibiting their development progress. This exhibited a distinct sequential ordering for these myogenic subpopulations. As shown in Figure 2A,B, we found that cells from the FB stage mainly consisted of myogenic progenitors, myoblasts and myocytes. However, the medium and late developmental stage cell types were mostly composed of different myofibers from the CB and AB stages. In addition, trajectory inference was strengthened by assessing the trends of myogenic gene expression across pseudotime with known myogenic biology (Figure 2C). Note that there was no expression of *PAX7* at the end stages of pseudotime, which is inconsistent with the consensus that *PAX7* is considered a marker for adult satellite cells.^25^ This is probably because of loss of transcriptional information from digestion of multinucleated muscle fibres or later nuclear extraction from *longissimus dorsi* tissues of adult cattle in the snRNA‐seq experimental procedure. For this reason, no satellite cells show up in our results.

To explore the molecular determinants of different cell states, we further performed gene expression analysis along the pseudotime trajectory and observed eight distinct gene clusters according to their expression pattern (Figure 2D,E). Gene clusters 1–8 including 286, 239, 268, 208, 161, 46, 262 and 23 genes, respectively (Table S1), whose expression patterns indicated that some genes increase or decrease monotonously with activation, while others exhibited non‐monotonic behaviour. For instance, most genes in gene cluster 1 increased monotonically across pseudotime, and we observed elevated expressions of *TPM3*, *TNNT1*, *MYH7*, *CSRP3*, *MUSTN1* and *NMRK2*. These genes enriched the GO–BP terms of ‘ATP metabolic process’, ‘muscle system process’, ‘striated muscle contraction’ and ‘muscle filament sliding’, suggesting that those genes might be associated with mature skeletal muscle fibres. In contrast, with most genes in clusters 3 and 7 decreased monotonically across pseudotime, they showed high‐level expression of *CENPF*, *NES*, *TPX2*, *UBE2C*, *CKAP2* and *HMGB2*, and enriched the GO–BP terms of ‘nuclear division’, ‘protein folding’, ‘RNA catabolic process’ and ‘protein targeting’, which might be involved in the development of foetal myogenic subpopulations such as myogenic progenitors and myoblasts. For gene clusters 2, 4–6 and 8, we observed non‐monotonic gene expression trends with high levels of *MEGF10*, *SORCS3*, *IGFBP6*, *HMCN2* and *SASH1*. Moreover, their enriched GO–BP terms such as ‘muscle fibre development’, ‘regulation of calcium‐mediated signalling’, ‘regulation of myoblast differentiation’ and ‘actin filament organization’ further suggested that these genes might be necessary to maintain normal skeletal muscle function and represent the forming of functional muscle fibres (Figure 2D,E). Thus we successfully characterized transcriptional programmes during myogenesis, whose monotonic and non‐monotonic temporal features suggested that myogenesis is a heterogeneous cellular process at multiple time points during development.

After recapitulating the skeletal muscle cell transcriptome landscape, SCENIC regulon inferring assays were applied successfully to identify specific transcriptional regulators associated with muscle development, and key TFs were identified in 18 clusters, as shown in Figure S1A. Here we focused on skeletal myogenic subpopulations, as shown in Figure S1B. The top 10 TF genes for each cluster are highlighted as well as some of their expression characteristics. The SCENIC analysis predicted that MSC, MYF5, MYOD1, FOXP3, ESRRA, BACH1, SIX2 and ATF4 might actively regulate the transcription of myogenic progenitors, myoblasts, myocytes, Type I fibres 1, Type I fibres 2, Type IIA fibres 1, Type IIA fibres 2 and Type IIX fibres, respectively (Figure S1B). Many of these interactions have been confirmed by numerous experiments, further strengthening the credibility of the predictions. Interestingly, myogenic progenitors and myoblasts share almost identical core TFs such as MSC, MYF5, GLI1, POLR3G, POLE3, POLE4, SETBP1 and RCOR1, while myocytes are specifically regulated by TFs such as TCF12, MYOD1, MYOG, WT1 and ZBTB18 (Figure S1B). This reflects the similarity in transcriptional regulation between myogenic progenitors and myoblasts, and specific transcriptional events might occur during myocyte formation. Notably, these specifically expressed TFs among neighbouring cell subpopulations are likely candidates for the promotion of cell fate transition, whose specific role can be the focus of future research.

### 3.3 RNA velocity analysis of transcriptional plasticity

**FIGURE 3:**
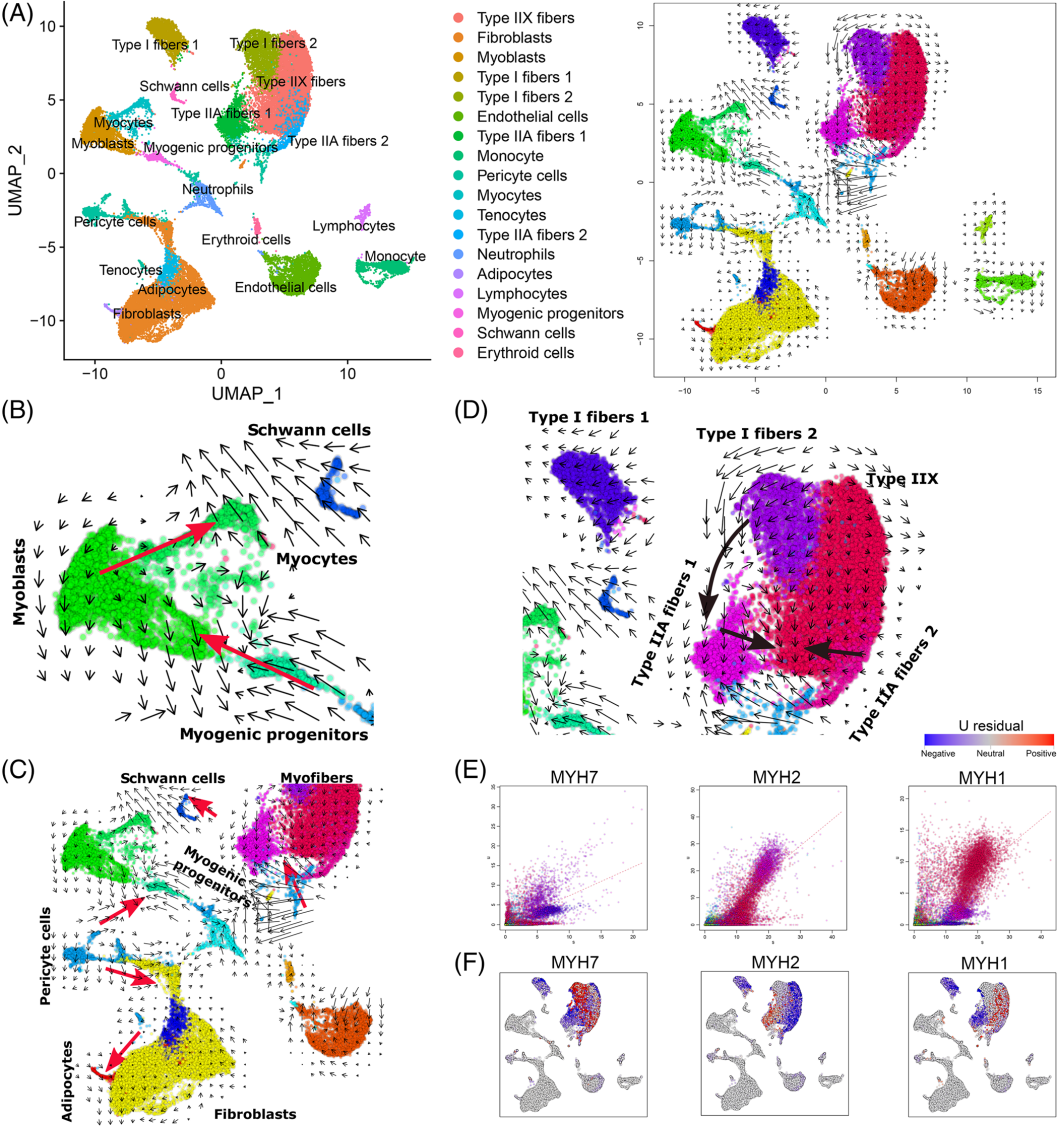
RNA velocity analysis unveils the dynamics of bovine myogenesis. (A) On the left is a UMAP plot of cells coloured by cell type; RNA velocity based on UMAP is visualized on the right, showing the landscape of bovine myogenesis. The arrows represent local average velocity on the vector field, whose direction indicates the fate of these cells, with their lengths reflecting the speed of differentiation events. The colours represent time points in development. (B) Velocities of myogenic progenitors, myoblasts, and myocytes shown on the UMAP plot in (A). (C) Commitment to pericyte fate. (D) Fate decision of myofibres. (E) Spliced–unspliced phase portrait underlying the velocity field of myofibers. The dashed line indicates the estimated slope (γ) fit for the developing cells. Cells above the γ fit line are genes induced, and those below indicate the repressed state. (F) Magnitude of the residuals for examples of marker genes of myofibers. The colour intensity corresponds to U residuals level, measuring the ratio between unspliced and spliced RNA. The negative U residuals refer to expected upregulation, and positive U residuals indicate expected upregulation of a gene. Marker genes are shown for Type I fibres (*MYH7*), Type IIA fibres (*MYH2*) and Type IIX fibres (*MYH1*).

As pseudotime analysis does not fully reveal the developmental dynamics of differentiation, we applied RNA velocity analysis to our scRNA‐Seq data set, which can be used to predict likely future transcriptional states of individual cells by distinguishing spliced and unspliced mRNAs. Here, RNA velocity based on UMAP plots was visualized; arrows each represent the local average velocity on a vector field with their length indicating the speed of the differentiation events (Figure 3A). As expected, the myogenic progenitors and myoblasts have RNA velocity vectors pointing towards the myocytes, suggesting strong myocyte lineage commitment. Intriguingly, most RNA velocity vectors within the myoblasts point backwards in pseudotime, indicating that myoblasts maintain the undifferentiated cell phenotype (Figure 3B). Significantly, Schwann cells and myocytes share similar trajectories with long RNA velocity vectors (Figure 3B). Schwann cells and myocytes are the core components of neuromuscular junctions, allowing the transmission of neuronal impulses to skeletal muscles for contraction.^26^ Moreover, we found that pericytes had the longest RNA velocity vectors pointing towards many cell clusters, such as myogenic progenitors, adipocytes, fibroblasts, myofibres and Schwann cells, suggesting that they have the capacity of multidirectional differentiation to maintain normal skeletal muscle development (Figure 3C).

The bovine *longissimus dorsi* is a fast‐twitch muscle mainly consisting of Type II fibres, and has a higher glycolytic capacity than slow‐twitch (Type I fibres).^27^ Previous reports have confirmed that fibre type switching such as I ↔ IIA ↔ IIX can be induced by exercise, by changes in neural activity, or under the influence of hormones during development.^28^ Consistent with this, RNA velocity analysis showed that Type II fibres are the majority of cells at the adult stage (Figure 1E), and RNA velocity vectors of Type I fibres 2 points towards Type IIA fibres 1. Moreover, Type IIA fibres 1 and Type IIA fibres 2 have RNA velocity vectors pointing towards the Type IIX fibres (Figure 3D–F). The heterogeneity of skeletal muscle fibres remains unknown, and understanding further details will elucidate mechanisms that control muscle development and function.

### 3.4 Ligand–receptor interaction prediction during bovine skeletal muscle development

**FIGURE 4:**
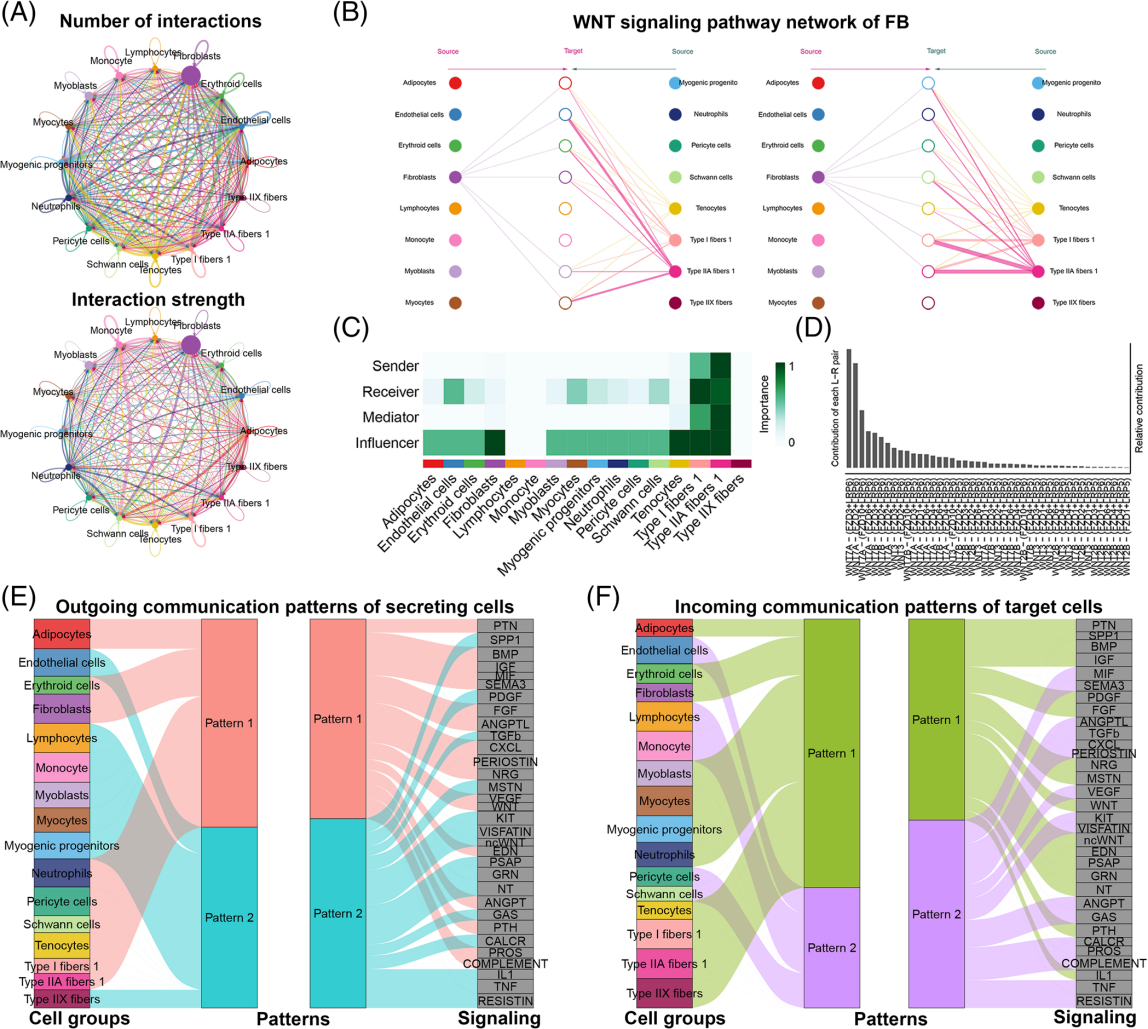
Capacity for intercellular communication among developing skeletal muscles. (A) Diagram of the ligand–receptor pairs at the FB stage. The number (up) and intensity (down) of cell interactions (ligand–receptor pairs) are presented generally, in which coloured dots indicate different cell groups, and the lines with arrows point to cell groups expressing the cognate receptors. The line thickness is proportional to the number (up) or strength (down) of ligand–receptor pairs, and the loops refer to autocrine circuits. (B) Hierarchical plot showing inferred cell interactions at the FB stage. Solid and open circles refer to the source and target, respectively. Lines represent intercellular interactions, and their thickness is proportional to the communication probability of the cell interaction. Line colours are consistent with the signalling source. (C) Heatmap shows the role (sender, receiver, mediator or influencer) of each cell group in WNT signalling at the FB stage. (D) Diagram of ligand–receptor pair contribution to WNT signalling pathway at the FB stage. (E) The outgoing communication patterns of secreting cells at the FB stage. The flow thickness represents the contribution of a signalling pathway to each mode of communication. (F) The inferred incoming communication patterns of target cells at the FB stage.

To define intercellular communication networks during bovine skeletal muscle development, we performed CellChat analysis on the scRNA‐seq data set from the FB, CB and AB stages, and dense networks were constructed consisting of 1122, 1257 and 920 significant cell interactions, respectively (*p* ≤ 0.05) (Figure 4A and Figure S3A,B). Our analysis then classified many ligand–receptor pairs among the 18 cell clusters, which were further categorized into significant signalling pathways, including BMP, IGF, WNT, MSTN, ANGPTL, TGFB, TNF, VEGF and FGF. Previous studies showed that signalling pathways activated by WNT ligands regulate myogenesis, so we examined specifically how WNT‐based communications change during bovine skeletal muscle development. At the FB stage, skeletal muscle fibres were the dominant source of WNT ligands, with minor contributions from fibroblasts (Figure 4B,C). Notably, The WNT ligand–receptor pairs WNT7A–FZD3/LRP6 were the major contributors to this communication network (Figure 4D), which is consistent with a report that WNT7A‐dependent expression is mediated by the canonical WNT cascade during embryonic myogenesis.^29^ Several studies have shown that WNT signalling plays a role in fibre type determination, so we have speculated the presence of cell–cell interactive mechanisms driving myofibre diversification during development.^30^ Compared with the FB stage, fibroblasts gained WNT responsiveness and myocytes became the most prominent source for WNT ligands at the CB stage (Figure S2A–C). Similar to the previous view that WNT signalling in mature skeletal muscle is poorly activated,^31^ myogenic progenitors emerged as the only sources of WNT signalling with significant autocrine signalling at the AB stage (Figure S2D–F). Multiple WNT ligand–receptor pairs are expressed at different time points during myogenesis and all of them, together with many other signalling pathways, are part of a complex regulatory network that drives the formation of skeletal muscle.

In another example at the three time points, monocytes or lymphocytes were the source of TNF ligands (Figure S3C), consistent with its multifunctional proinflammatory cytokine role secreted predominantly by these cell types.^32^ Network centrality analysis of the VEGF signalling pathway network confirmed adipocytes and fibroblasts were the dominant source of VEGF ligands acting on endothelial cells, with minor contributions from skeletal muscle cell populations. However, skeletal muscle fibres gained VEGF responsiveness as the body developed (Figure S3D). Moreover, CellChat analysis predicted that the FGF signalling pathway network is complex and highly redundant with multiple ligand sources, and adipocytes and fibroblasts demonstrated significant autocrine signalling (Figure S3E).

To explore how multiple cell groups and signalling pathways coordinate to function during myogenesis, CellChat pattern recognition modules were applied and showed that certain cell types simultaneously activate multiple signalling pathways and rely on largely overlapping incoming and outgoing signalling networks at different developmental nodes. For example, the application of this analysis revealed two, three, and four patterns for outgoing signalling and incoming signalling at the FB, CB and AB stages, respectively (Figure 4E,F and Figure S3F). The patterns here represent multiple pathways that change over time, notably, adipocytes share the same pattern 1 with fibroblasts for both incoming and outgoing signalling at different stages. Thus, multiple cell types in skeletal muscle contribute to support complex signal transduction, in which adipocytes and fibroblasts seem to play key roles. Moreover, outgoing and incoming signalling patterns also provide specific autocrine and paracrine signalling pathways for a given cell type. For instance, at the FB stage, the major autocrine pathways between adipocytes are PTN, BMP, IGF and FGF, while the major paracrine signalling pathways for adipocytes are SPP1, PDGF and MSTN (Figure 4E,F). These analyses can potentially help in the deeper understanding of regulatory mechanisms driving differentiation of the skeletal muscle lineage.

### 3.5 scATAC‐seq reveals chromatin accessibility landscapes of the bovine skeletal muscle

**FIGURE 5:**
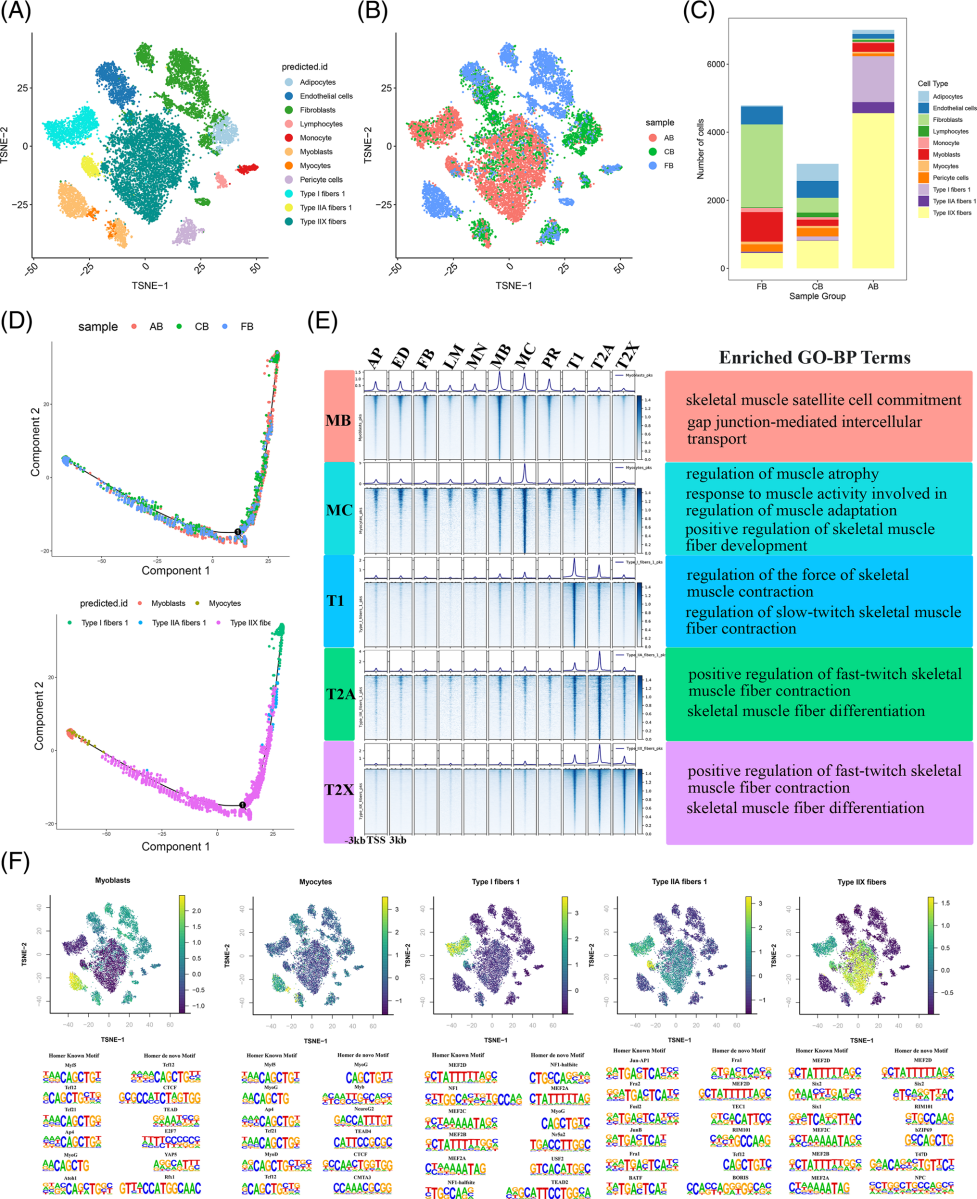
A single‐cell epigenetic atlas of bovine skeletal muscle. (A, B) TSNE representation of the scATAC‐seq data. TSNE plot of cells coloured by cell type (A) and the developmental time point (B). (C) The percentage of different cell types classified by scATAC‐Seq. (D) Reconstructed trajectory of bovine skeletal myogenic subpopulations with Monocle2. The trajectory plot indicates the direction of developmental time point (up) and cell types (down) were assigned by different colours and arranged by pseudotime. (E) GO–BP results of genes potentially regulated by skeletal myogenic subpopulations at specific open regions. The heatmaps on the left show ATAC‐seq signals around peak centres in skeletal muscle cell types, indicating open chromatin, respectively, in MB, MC, T1, T2A and T2X. Key: MB, myoblast; MC, myocyte; T1, Type I fibres; T2A, Type IIA fibres; T2X, Type IIX fibres. (F) Motifs and their TF sets were predicted in each skeletal myogenic subpopulations.

Our scRNA‐seq results focus on RNA expression, ignoring the epigenetic changes which are the primary determinants of cellular potential. To gain more insights into the development of bovine skeletal muscle at the chromatin level, we obtained chromatin accessibility profiles of 24,333 cells from these successive developmental stages (7274, 7433 and 9626 single cells) by scATAC‐seq (Figure 1A). The library quality was evaluated based on the insertion lengths and peak signal distributions, which passed the assessment (Figure S4A–C). There were only 11 cell types identified at the three developmental stages by merging samples and eliminating batch effects (Figure 5A,B). Population label of the scATAC‐seq populations showed good agreement with that of scRNA‐seq populations (Figure 5C). Because chromatin accessibility in regulatory regions precedes gene activity, it may determine the future direction of gene transcription. Previous research showed that specific chromatin regulators maintain a globally open chromatin state for gene transcriptional activation; and some factors can cause specific local gene silencing until the activation of cell differentiation.^33^ To investigate whether there is an overall dynamic change in the accessibility pattern associated with bovine skeletal muscle development, we performed pseudotime analysis of scATAC‐seq data using the same method as our previous scRNA‐seq, described above. The sequential ordering for these myogenic subpopulations in the scATAC‐seq results was highly compatible with those from the scRNA‐seq data in general (Figure 5D). Along the inferred trajectory, we observed dynamic changes in the accessibility of lineage‐specific genes, forming eight clusters (Figure S4D). Gene clusters 1–8 consist of 555, 316, 825, 526, 488, 227, 539 and 200 genes, respectively, with monotonic and non‐monotonic gene activity patterns, consistent with the gene expression pattern obtained by scRNA‐seq (Table S2, Figure S4D).

Next, we invoked and visualized cell type‐specific peaks to identify potential drivers of the skeletal muscle transcriptome, and genes that potentially regulated by cell type‐specific open regions were annotated functionally to determine biological processes associated with these elements (Figure 5E, Figure S4E). From these GO–BP results, we found terms, such as ‘skeletal muscle satellite cell commitment’, ‘response to muscle activity involved in regulation of muscle adaptation’, ‘regulation of slow‐twitch skeletal muscle fibre contraction’, ‘positive regulation of fast‐twitch skeletal muscle fibre contraction’ and ‘skeletal muscle fibre differentiation’, were enriched in peaks from myoblasts, myocytes, Type I fibres, Type IIA fibres and Type IIX fibres, respectively, which are generally consistent with the future direction of gene transcription in myogenesis (Figure 5E). Because cell type‐specific peaks likely harboured motifs allowing for the binding of TFs, using HOMER, we further performed motif enrichment analysis for cell type‐specific peaks to predict TF sets controlling of gene expression in skeletal muscle (Figure 5F, Figure S4F). Finally, large sets of TFs, both known and novel, were predicted for each cell type.

**FIGURE 6:**
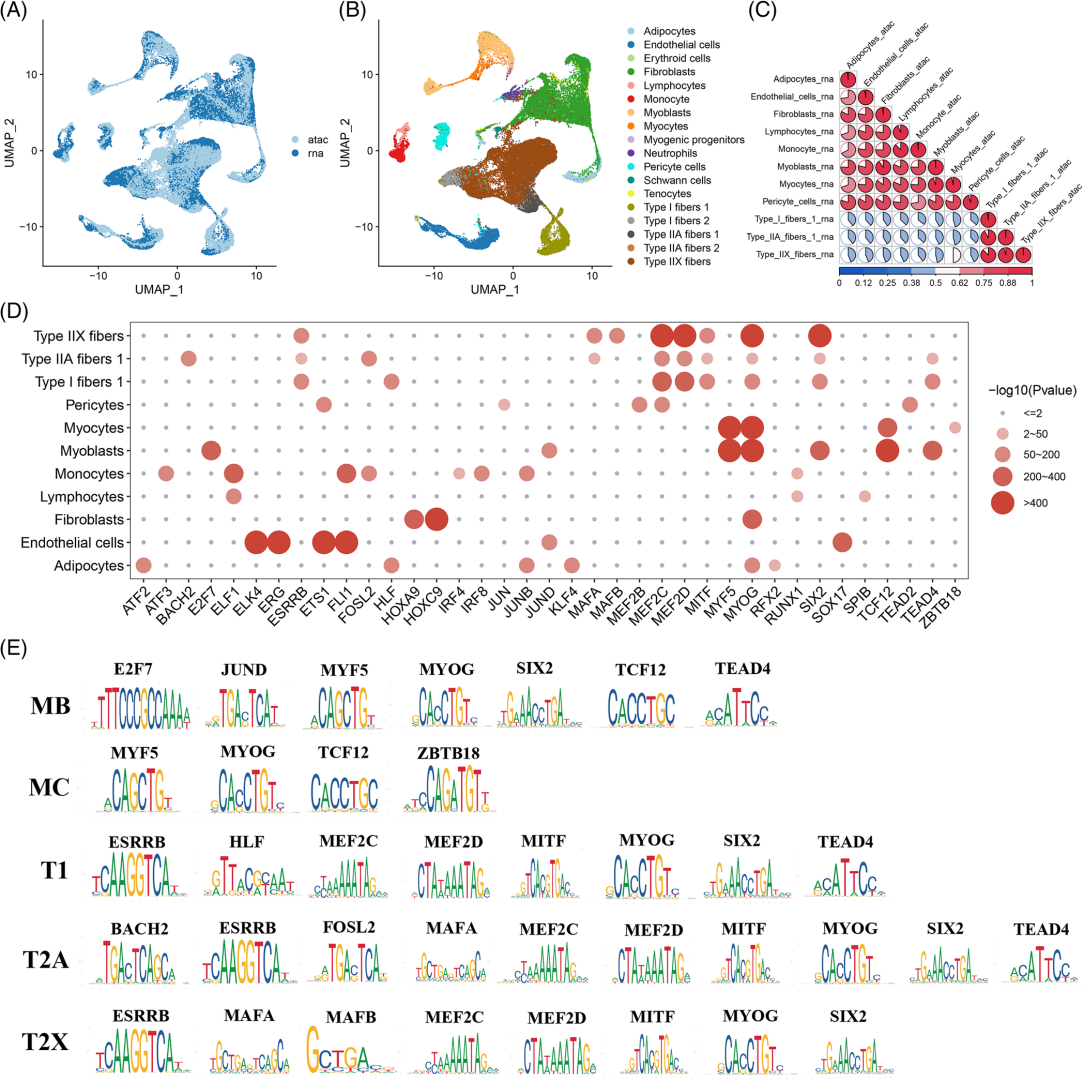
Integrated analysis of scRNA‐seq and scATAC‐seq results. (A, B) Joint UMAP visualization of scRNA‐seq and scATAC‐seq data sets. Cells are coloured by modality (A) and predicted cell types (B). (C) Correlation matrix showing the unbiased and pairwise comparison of skeletal myogenic subpopulations based on scRNA‐seq and scATAC‐seq results. The colour bar indicates Pearson correlation coefficient strength. (D) Dotplot of significantly shared TFs from SCENIC regulon inferring assay and HOMER analysis. (E) Transcription factor motifs significantly enriched in each skeletal myogenic subpopulations.

To further explore the relationship between regulation of the expression of some critical genes and chromatin accessibility, we successfully integrated our scRNA‐seq and scATAC‐seq results (Figure 6A,B). Overall, these two modalities were validated correlated by examining markers in each of the annotated cell types (Figure 6C), though existing differences were caused by experimental and threshold deviations between the scRNA‐seq and scATAC‐seq atlases. Comparing potential TFs from a previous SCENIC regulon‐inferring assay and HOMER analysis, significantly shared TFs were revealed, including associated TFs controlling the transcriptional responses of skeletal muscle resident cell types in myogenesis (Figure 6D,E). For instance, the top enriched motifs included binding sites for E2F7, JUND, MYF5, MYOG, SIX2, TCF12 and TEAD4 in myoblasts, and the top enriched motifs in myocytes contained binding sites for MYF5, MYOG, TCF12 and ZBTB18. Among these muscle‐enriched TFs, frequent binding sites for MYF5 and MYOG have been studied widely in the context of myogenesis,^4^ while sites for TFs such as E2F7, JUND and ZBTB18 have not attracted much attention. Moreover, our results suggested specific roles for HLF, BACH2, FOSL2, MAFA and MAFB in gene activation during fibre‐type formation. Overall, gene expression is a dynamic process generally regulated by cell type‐specific TFs interacting with defined motifs, and these specifically expressed TFs are likely to be candidates for the promotion of cell fate transition, whose importance in myogenesis can be the focus of future studies.

## 4 DISCUSSION

Studying developmental biology, where individual cells determine their fate, and defining the lineage, proportions, and molecular characteristics of different cell types is fundamental to our understanding. Large‐scale single‐cell analysis allows us to explore the identity of an individual cell and the factors underlying it in a data‐driven way. A recent study provided a scenario for human limb myogenesis from early embryo to adulthood in vivo,^6^ yet myogenic development in bovine tissues at the single‐cell level is poorly understood. Here, we used scRNA‐seq/scATAC‐seq analyses to establish a complete atlas of transcriptome programmes and chromatin landscape of the different cell types present in bovine skeletal muscles from early embryonic to postnatal periods. To our knowledge, this study for the first time has revealed a single‐cell dynamic chromatin/transcription landscape for normal bovine skeletal muscle development.

Although genetic studies in recent decades have identified key regulators of skeletal muscle ontogeny successfully, our knowledge on the architecture of their gene regulatory network and dynamic changes driving cell fate transitions is still fragmentary. Using computational approaches, we inferred the developmental dynamic trajectories of the developing bovine skeletal muscle, in which functionally‐distinct myogenic subpopulations were identified within the various stages and the relative transcriptional changes determining the differentiation trajectory were described accurately. For example, we found that myogenic progenitors and myoblasts at this stage are widely enriched with cell cycle regulatory genes such as *CENPF*, *TPX2* and *CKAP2*.^34^, ^35^, ^36^ For these intermediate state cells, enriched GO–BP terms such as ‘regulation of myoblast differentiation’ and ‘muscle fibre development’ further helped in deciphering the molecular events, corresponding to postnatal muscle formation. At the end of the pseudotime trajectory, genes associated with basic structure and function of mature skeletal muscle, such as *TPM3*, *TNNT1* and *MYH7*,^37^, ^38^, ^39^ were highly expressed in gene cluster 1, as well as *CSRP3* and *MUSTN1*, known to be important regulatory factors acting during skeletal muscle differentiation.^40^, ^41^ It would be interesting to explore the function of these genes in future studies.

As already mentioned, single‐cell molecular profiling has problems associated with static measurements; however, temporal information is essential to decipher the complex developmental processes of skeletal muscle. For inferring future cellular states, we applied an RNA velocity framework to a bovine skeletal myogenesis atlas, based on a well‐defined model of the transcription process. Overall, the majority of inferred differentiation paths agreed with our current understanding of the developmental biology of skeletal muscle. In particular, the differentiation trend of myogenic subpopulations (myogenic progenitors → myoblasts → myocytes, and Types I → IIA → IIX) were shown from the inferred pseudotime trajectory and from the calculated RNA velocity. Of note, when applying the RNA velocity framework, intron retention events associated with splicing heterogeneity were not considered in our modelling.

In multicellular organisms, the formation of highly differentiated tissues is determined by an intricate network of cells with particular biological functions to a great extent, and fine‐tuned communication among these different cells is critical for maintaining the homeostasis and function of tissues. Specifically, cell‐to‐cell interactions can be realized in autocrine or paracrine manners.^42^ Furthermore, in a local environment, extracellular signalling of the interactions between secreted ligands and cell surface receptors can generate multiple cell fate decisions.^43^ It is important to note that an increasing number of studies have inferred cell‐to‐cell communications by assessing the gene expression levels of receptor–ligand pairs across cell populations and uncovered meaningful biological insights. For instance, Shang et al. proposed several putative ligand–receptor interactions in human early neural differentiation, reflecting the identity of each cell subpopulation possessing a distinctive spectrum of ligands and receptors associated with the process. This facilitated a deeper understanding of the cell fate decision and regulatory mechanisms driving the differentiation of the neural lineage.^44^ Additionally, a dense ligand–receptor network facilitating extensive communication was observed between diverse mouse heart cell types, which revealed prevalent sexual dimorphism in gene expression levels.^45^ Here, we used ligand–receptor interaction modelling to identify new regulatory programmes among distinct cell populations in skeletal muscle, in which different cell types showed a certain degree of specificity in their ligand–receptor spectrum. For instance, fibroblasts and adipocytes showed the strongest intercellular relationships among multiple cell populations, while the weakest cell communications were observed among myogenic cells. These specific ligand–receptor pairs might reveal the unique regulatory modes of different cell types in skeletal muscle. Furthermore, ligand–receptor interactions also occur at the protein level, and adding this information for individual cells could further enhance the modelling accuracy of CellChat.

Generally, gene expression is regulated by complex interplays between TFs and cis‐regulatory DNA elements, and assessing genome‐wide chromatin accessibility can facilitate the identification of these functional regions.^46^ Single‐cell technologies offer new perspectives to study the mechanisms underlying cell properties, in which scRNA‐seq provides transcriptional landscapes measuring gene expression in each cell, while scATAC‐seq serves as a metric of chromatin accessibility to contribute insight into cellular transcriptional heterogeneity.^47^ Here, we investigated the changes in the epigenetic landscape by performing scATAC‐seq in developing bovine skeletal muscle, and integration of the scRNA‐seq and scATAC‐seq results further provided a comprehensive view of the cellular regulatory state. As expected, we revealed specific TFs, such as E2F7, JUND, ZBTB18, HLF, BACH2, FOSL2 and MAFA, that might regulate cell fate transition during myogenesis. In addition, with the exception of some cell‐specific TFs, many were also coexpressed among cell clusters, further supporting the previous consensus that genes in different cell subsets are expressed in a partially overlapping dynamic manner to form a complex network of interconnections during skeletal myogenesis.^48^ Therefore, these findings are potentially valuable in advancing our current understanding of critical genetic regulatory networks associated with bovine skeletal muscle development, and our future work will focus on deciphering the functional importance of these candidate TFs and their target genes.

## AUTHOR CONTRIBUTIONS

Jincheng Zhong, Ruihua Dang, Cuicui Cai and Binglin Yue conceived the ideas and designed the work. Cuicui Cai and Binglin Yue performed the majority of analysis with contributions from Hui Wang, Xin Cai, Jiabo Wang, Zhixin Chai, Jikun Wang, Haibo Wang, Ming Zhang, Nan Yang, Zhijuan Wu. Xueyao Yang, Peng Wan and Yulian Li prepared the samples. Binglin Yue wrote the manuscript. Jiangjiang Zhu revised the manuscript. All authors have read and approved the final manuscript.

## CONFLICT OF INTEREST STATEMENT

The authors declare no conflict of interest.

## Supporting information


**FIGURE S1.** Result of SCENIC regulon inferring assays.
(A) SCENIC binary heatmap depicting enriched regulons of each cell. The regulon represents regulatory network of TFs and their binding motifs, with black blocks indicating cells that are active. The row indicates regulons while the column depicts a single cell.
(B) The top 10 regulons in each bovine skeletal myogenic subtype are highlighted in red, and the representative TFs and their motifs are listed in the right panel.

**FIGURE S2.** The inferred WNT signalling networks at the CB and AB stages.
(A) Hierarchical plot showing inferred cell interactions at the CB stage.
(B) Heatmap shows the role (sender, receiver, mediator, or influencer) of each cell group in WNT signaling at the CB stage.
(C) Diagram of ligand–receptor pair contribution to WNT signaling pathway at the CB stage.
(D) Hierarchical plot showing inferred cell interactions at the AB stage.
(E) Heatmap shows the role (sender, receiver, mediator, or influencer) of each cell group in WNT signaling at the AB stage.
(F) Diagram of ligand–receptor pair contribution to WNT signaling pathway at the AB stage.

**FIGURE S3.** CellChat analysis of intercellular communication among developing skeletal muscles.
(A) Diagram of the ligand–receptor pairs at the CB stage.
(B) Diagram of the ligand–receptor pairs at the AB stage.
(C) The inferred TNF signaling networks at the FB, CB, and AB stages.
(D) The inferred VEGF signaling networks at the FB, CB, and AB stages.
(E) The inferred FGF signaling networks at the FB, CB, and AB stages.
(F) The outgoing communication patterns of secreting cells at the CB and AB stages (up), and the inferred incoming communication patterns of target cells at the CB and AB stages (down).

**FIGURE S4.** Additional information for Figure 5.
(A–C) Transcription start site enrichment plot (left) and typical fragment size distribution (right) of the FB (A), CB (B), and AB (C) stage scATAC‐Seq results, respectively.
(D) Eight clusters of pseudotime gene activity are clustered hierarchically during bovine myogenesis.
(E) GO–BP results of genes that are potentially regulated by other cell types at specific open regions. Key: AP, adipocytes; ED, endothelial cells; FB, fibroblasts; LM, lymphocytes; MN, monocytes; PR, pericytes.
(F) Motifs and their TF sets were predicted for other cell types.

**TABLE S1.**Supporting Information.

**TABLE S2.** Supporting Information.

## Data Availability

The single‐cell sequencing data used in this research is deposited in CNCB GSA databases under accession number: CRA006626.
