# Supplementary figures and images for: Transcriptional and open chromatin analysis of bovine skeletal muscle development by single‐cell sequencing
Source: Cell Prolif. 2023 Mar 1;56(9):e13430. doi: 10.1111/cpr.13430 (PMC10472525; doi:10.1111/cpr.13430)

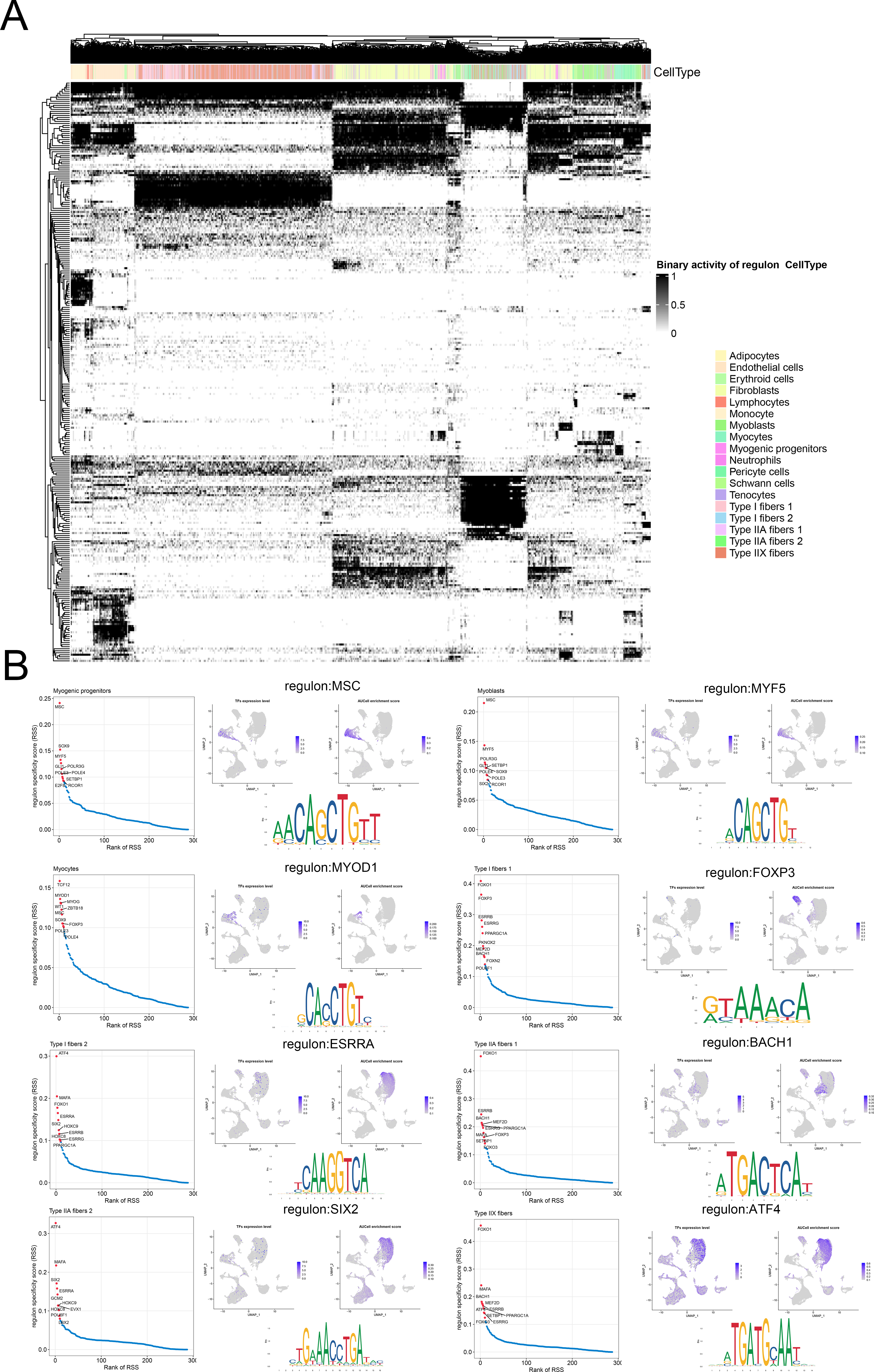

Supplement: Supplementary file 1 — FIGURE S1. Result of SCENIC regulon inferring assays. (A) SCENIC binary heatmap depicting enriched regulons of each cell. The regulon represents regulatory network of TFs and their binding motifs, with black blocks indicating cells that are active. The row indicates regulons while the column depicts a single cell. (B) The top 10 regulons in each bovine skeletal myogenic subtype are highlighted in red, and the representative TFs and their motifs are listed in the right panel. [file CPR-56-e13430-s002.tif]

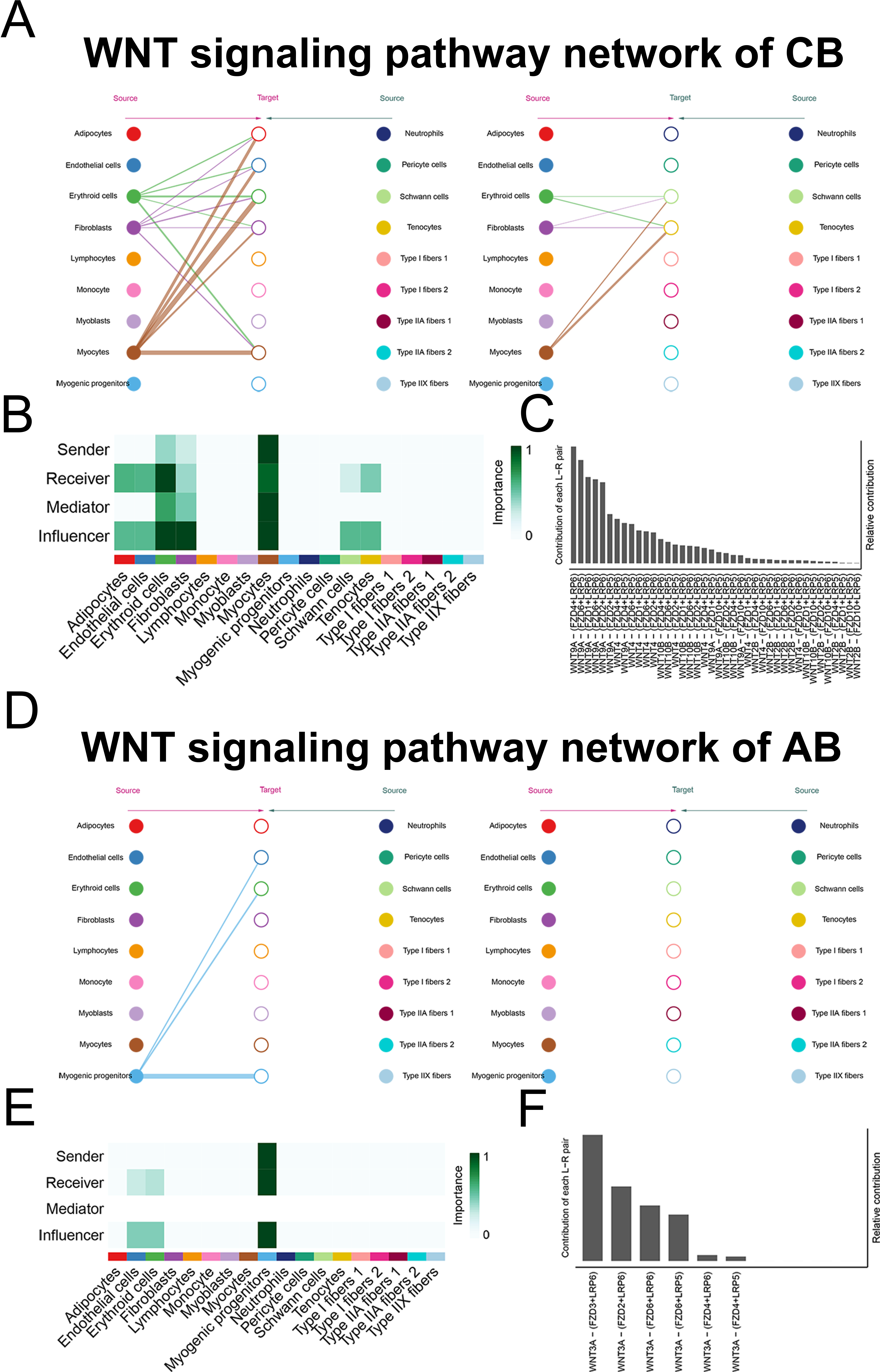

Supplement: Supplementary file 2 — FIGURE S2. The inferred WNT signalling networks at the CB and AB stages. (A) Hierarchical plot showing inferred cell interactions at the CB stage. (B) Heatmap shows the role (sender, receiver, mediator, or influencer) of each cell group in WNT signaling at the CB stage. (C) Diagram of ligand–receptor pair contribution to WNT signaling pathway at the CB stage. (D) Hierarchical plot showing inferred cell interactions at the AB stage. (E) Heatmap shows the role (sender, receiver, mediator, or influencer) of each cell group in WNT signaling at the AB stage. (F) Diagram of ligand–receptor pair contribution to WNT signaling pathway at the AB stage. [file CPR-56-e13430-s006.tif]

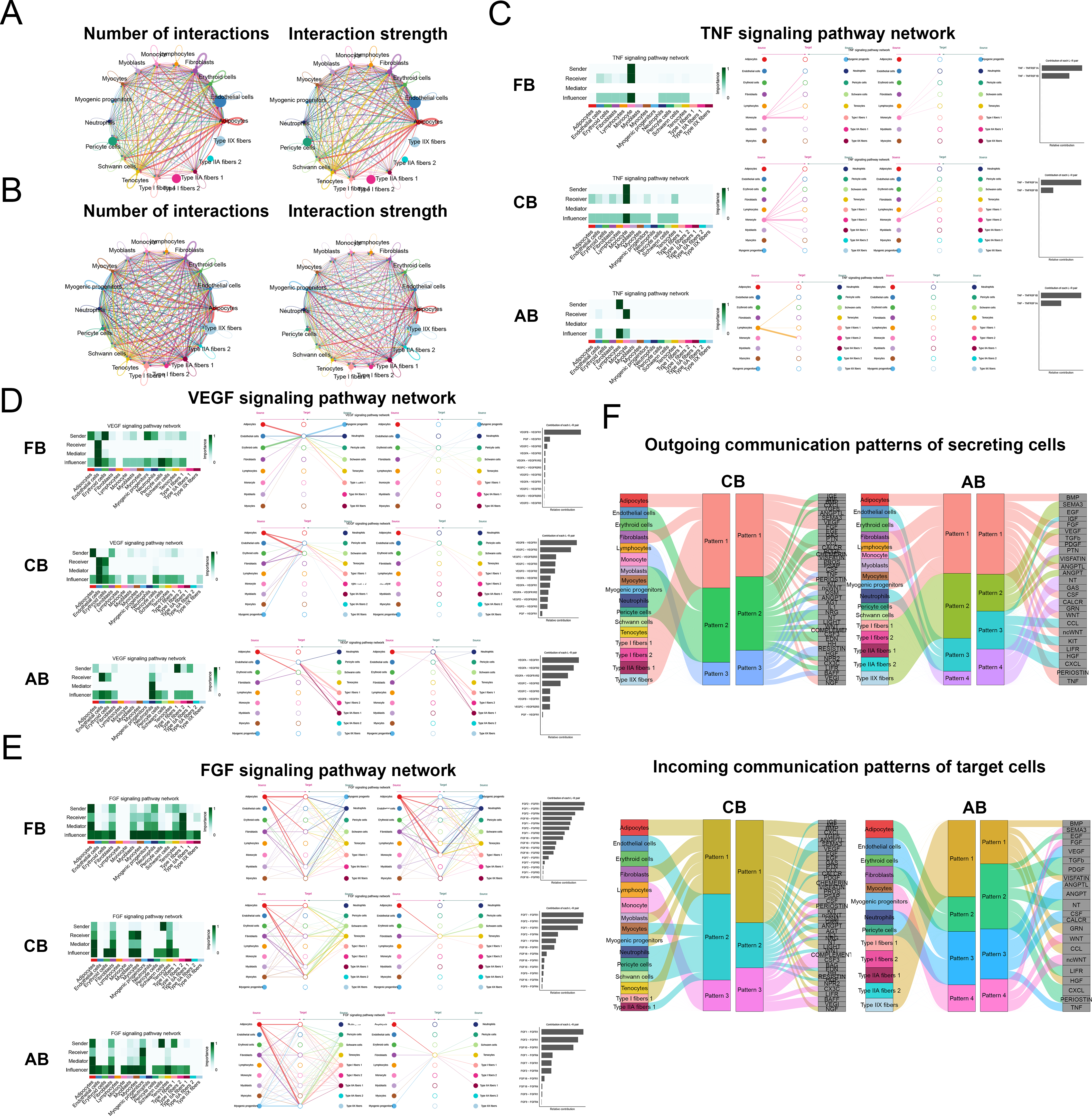

Supplement: Supplementary file 3 — FIGURE S3. CellChat analysis of intercellular communication among developing skeletal muscles. (A) Diagram of the ligand–receptor pairs at the CB stage. (B) Diagram of the ligand–receptor pairs at the AB stage. (C) The inferred TNF signaling networks at the FB, CB, and AB stages. (D) The inferred VEGF signaling networks at the FB, CB, and AB stages. (E) The inferred FGF signaling networks at the FB, CB, and AB stages. (F) The outgoing communication patterns of secreting cells at the CB and AB stages (up), and the inferred incoming communication patterns of target cells at the CB and AB stages (down). [file CPR-56-e13430-s004.tif]

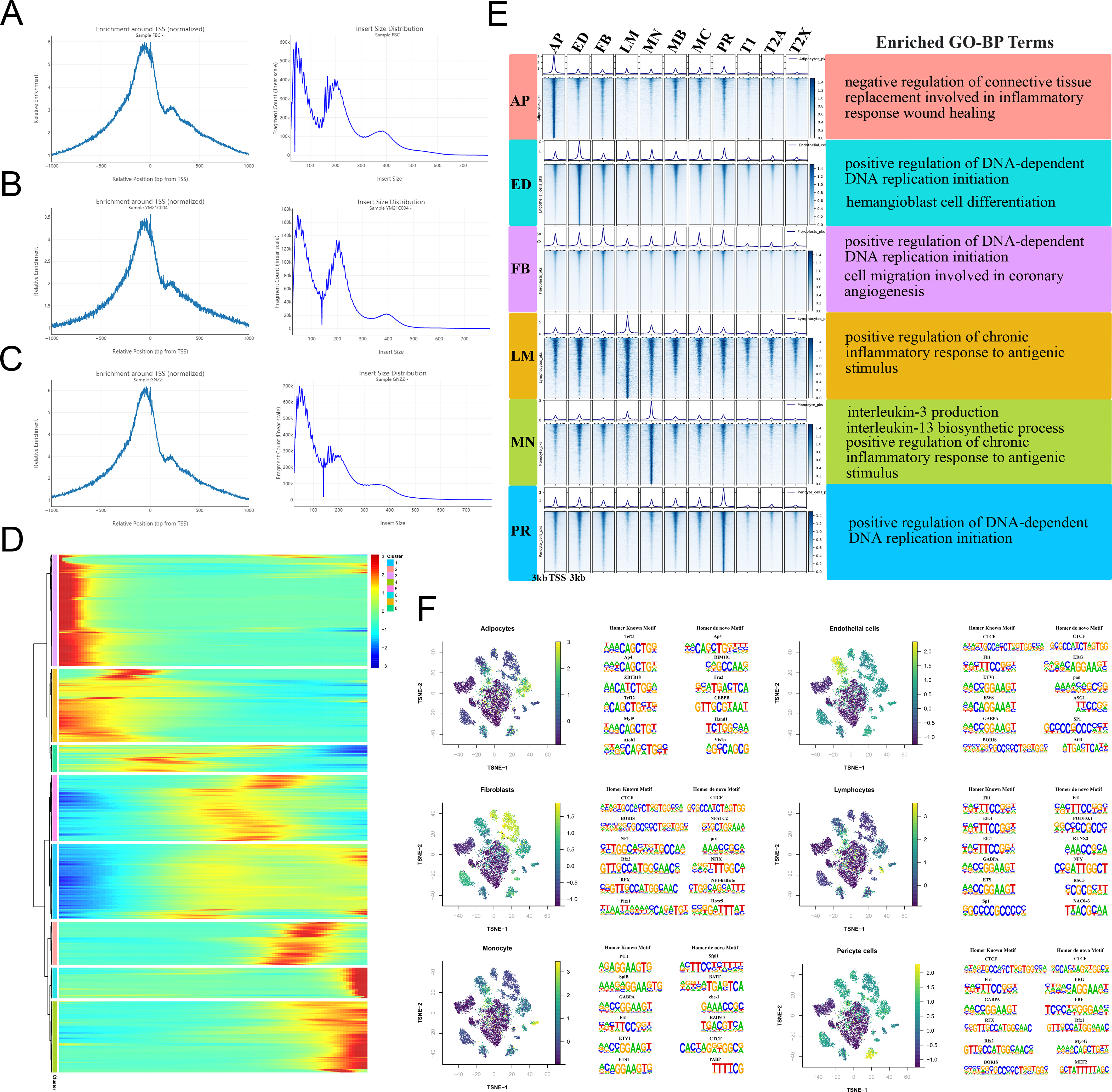

Supplement: Supplementary file 4 — FIGURE S4. Additional information for Figure 5. (A–C) Transcription start site enrichment plot (left) and typical fragment size distribution (right) of the FB (A), CB (B), and AB (C) stage scATAC‐Seq results, respectively. (D) Eight clusters of pseudotime gene activity are clustered hierarchically during bovine myogenesis. (E) GO–BP results of genes that are potentially regulated by other cell types at specific open regions. Key: AP, adipocytes; ED, endothelial cells; FB, fibroblasts; LM, lymphocytes; MN, monocytes; PR, pericytes. (F) Motifs and their TF sets were predicted for other cell types. [file CPR-56-e13430-s003.tif]
